# Antibiotic Consumption and Microbiological Epidemiology in Surgery Departments: Results from a Single Study Center

**DOI:** 10.3390/antibiotics9020081

**Published:** 2020-02-13

**Authors:** Dana Carmen Zaha, Simona Bungau, Diana Uivarosan, Delia Mirela Tit, Teodor Andrei Maghiar, Octavian Maghiar, Carmen Pantis, Ovidiu Fratila, Marius Rus, Cosmin Mihai Vesa

**Affiliations:** 1 Department of Preclinical Disciplines, Faculty of Medicine and Pharmacy, University of Oradea, 1 December Sq., 410081 Oradea, Romania; danaczaha@gmail.com (D.C.Z.); diana.uivarosan@gmail.com (D.U.); v_cosmin_15@yahoo.com (C.M.V.); 2 Department of Pharmacy, Faculty of Medicine and Pharmacy, University of Oradea, 29 N. Jiga St., 410028 Oradea, Romania; mirela_tit@yahoo.com; 3 Department of Surgical Disciplines, Faculty of Medicine and Pharmacy, University of Oradea, 1 December Sq., 410081 Oradea, Romania; teodormaghiar@yahoo.com (T.A.M.); octimaghiar@yahoo.com (O.M.); pantisc@yahoo.com (C.P.); 4 Department of Medical Disciplines, Faculty of Medicine and Pharmacy, University of Oradea, 1 December Sq., 410081 Oradea, Romania; ovidiufr@yahoo.co.uk (O.F.); rusmariusr@yahoo.com (M.R.)

**Keywords:** antibiotics, pathogens, surgery, dose, bacteria, susceptibility

## Abstract

The spectrum and antibiotic sensitivity of isolated strains vary between departments, hospitals, countries; the discrepancies are related to the use and dosage of these antibiotics. The purpose of our research was to compare the type of pathogens and the susceptibility of the isolated strains, as well as the use of antibiotics in the surgical departments of the Emergency Clinical County Hospital, Oradea, Romania; for one year, all the patients admitted to the mentioned sections were monitored. Antibiotic sensitivity of isolated strains was expressed using cumulative antibiogram. The total consumption of antibiotics was 479.18 DDD/1000 patient-days in the surgical sections. The most commonly used drugs were cephalosporins third and first generation, and clindamycin. Infections of wounds, urinary tract and fluids were most commonly diagnosed, and the most isolated was *Escherichia coli*, followed by *Staphylococcus aureus* and *Enterococcus faecalis*. The most commonly prescribed antimicrobial was ceftriaxone, but its sensitivity was low. This study revealed that the intake of antimicrobials in the surgical sections is increased and the comparison of antimicrobial prescriptions, sensitivity rates, and the spectrum of isolated pathogens showed differences between antimicrobials.

## 1. Introduction

Surgical site infection is a major complication of operative procedures, and hospital-acquired infections contribute to morbidity, mortality and healthcare costs. Surgical antibiotic prophylaxis is a solution for preventing the postoperative infections if appropriate antibiotics, dose, durations, surgical procedures are given at the correct time, and it should cover the likely pathogens. Previous studies of antibiotic prophylaxis usage have shown wide variation in selection, timing and duration [1]. Antibiotic prophylaxis is the standard measure for contaminated and clean-contaminated surgery, and for surgery involving the insertion of artificial devices. Less accepted indications for prophylaxis include clean operations in patients with impaired host immunity or patients from neurosurgery, open heart surgery and ophthalmic surgery. Surgical antibiotic prophylaxis protocols in hospitals must be regularly reviewed considering also the cost of antibiotics therapy, the waste generated by antibiotics consumption [2,3], and the endemicity of pathogens, including colonization especially multidrug-resistant bacteria. Appropriate antibiotic prophylaxis is able to reduce the risk of postoperative infections, but in the same time, antibiotic use increases the selective pressure to arise antimicrobial resistance [4]. 

A special group of pathogens called ESKAPE (*Enterobacter* spp., *Staphylococcus aureus*, *Klebsiella* spp., *Acinetobacter baumannii*, *P. aeruginosa*, *Enterococcus* spp.) is the cause of most healthcare-associated infections and demonstrate concerning patterns of antibiotic resistance and the patients’ colonization by these strains is not negligible at all [5,6]. The European Centre for Disease Prevention and Control (ECDC) has confirmed that the trend in some European countries, including Romania is the increase of antimicrobial resistance even though not all hospitals reported it. The increasing trend in the resistance of gram-negative species is more concerning, while the resistance of gram-positive bacteria remains almost the same [7]. In Romania, in 2017, the reported resistance of *K. pneumoniae* to third generation cephalosporins was 64.1%, 22.5% to carbapenems, 31.4% to fluoroquinolones and 58.6% to aminoglycosides corresponding to a combined resistance of 55.4%. *P. aeruginosa* presented rates of 55.9%, 53.4% resistance to piperacillin-tazobactam, 62.1% to fluoroquinolones and a combined resistance equal to 59.1% [7]. Public health attention is required in order to identify the infections and limit the transmission of *C. difficile*, carbapenem-resistant, and the extended-spectrum beta lactamase (ESBL) producing Enterobacterales, vancomycin resistant *Enterococcus*. 

Several studies have demonstrated that monitoring the hospital prescriptions of antimicrobials can help to understanding the relationship between antibiotics use and development of resistance in pathogens for specific patient care areas [8,9]. Increases in the prevalence of resistant pathogens in hospitals could be explained by the high selective pressure of antimicrobials commonly used in hospitalized patients. Therefore, it is essential to have surveillance data on antimicrobial resistance and antibiotic consumption in the hospital/community. Antimicrobials are the second most commonly prescribed drugs in the world next to cardiovascular drugs. Those used in hospitals vary widely, and there is a large variability in the spectrum and sensitivity to antibiotics of isolated strains. To express sensitivity of strains to antibiotics, a good tool is a cumulative antibiogram; this report is also used for initial empirical antibiotic therapy in the management of infections in patients whose microbiological tests show no available treatment options.

In order to provide possible future intervention for clinically rational use of antibiotics, the variability and spectrum of the sensitivity of the isolated pathogens and prescribing patterns of antimicrobials were analyzed on the surgical departments of the studied hospital. 

## 2. Results

In 2017, the overall antimicrobial use at the hospital expressed as the total number of DDD of antibiotics was 114,269, and the overall number of administered defined daily doses (DDDs) per 1000 PD was 2180.4. Considering DDDs, the surgical wards occupy the first place, with a value of 53,387. Dividing antibiotic use (expressed as DDDs) by the number of patient-days, it was found that a quarter of the total antimicrobials were consumed on surgical wards (479.18 DDD/1000 PD).

**Figure 1:**
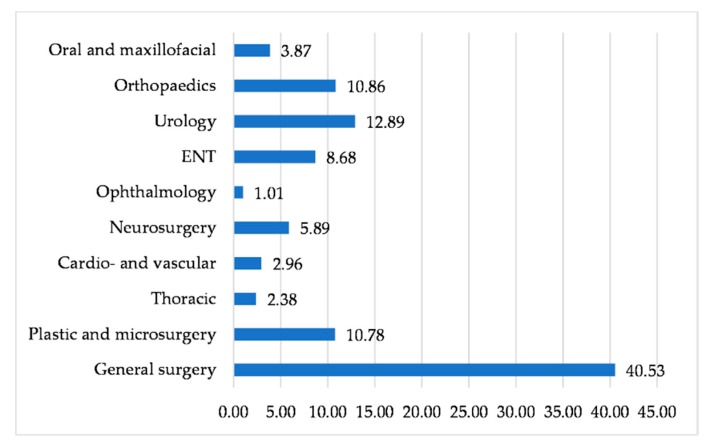
Antimicrobials prescribed by surgical departments expressed as DDDs percentages.

The parenteral administration of antibiotics represented almost 90.26% (432.39 DDD/1000 PD), while oral forms only 9.76% (46.79 DDD/1000 PD) of the total antibiotic intake. Antimicrobials use differed according to the type of surgical ward; most antibiotics were prescribed in the following departments: General surgery, urology, orthopedics, burns and plastic surgery (Figure 1). 

The pattern of antibiotic use by class showed that the most frequently prescribed antibiotics in all departments of the hospital were cephalosporins (54.30%), followed by fluoroquinolones (10.99%), penicillin and beta-lactamase inhibitors mixtures (10.76%), aminoglycosides (7.65%), carbapenems (5.46%). Less frequently prescribed in the whole hospital were linezolid, macrolides and tetracyclines (Table 1). 

Cephalosporins, including combinations (ATC group J01D) were the most widely used antibiotics, with the total DDD/1000 PD of 314.29 (65.02%) followed by penicillin and beta-lactamase inhibitors combinations (13.73%), fluoroquinolones (7.57%), aminoglycosides (5.79%), and clindamycin (5.07%). Colistin, glycol-peptides, carbapenems, tigecycline, linezolid, macrolides and tetracycline were less administrated to surgical patients.

The total consumption of antimicrobials was 479.18 DDD/1000 PD. Table 2 shows the proportion of different types of systemic antibiotics consumption according to the ATC classification.

In all surgical wards, it was noticed a high consumption of third generation cephalosporins (ceftriaxone, ATC group J01DD04) and ceftazidime (ATC group J01DD02) representing more than half of prescriptions. The first-generation cephalosporin (cefuroxime, ATC group J01DC02) still represented higher consumption of the total inpatient antimicrobials. Clindamycin (ATC group J01FF01) was included in the five most-used antibiotics (with a rate of 24.22 DDD/1000 PD), followed by gentamycin (ATC group J01GB03) (with a rate of 22.79 DDD/1000 PD). In the studied surgical inpatients, the lower DDD/1000 PD was reported for polymyxins (0.10), linezolid (0.08), macrolides (0.05) as well for vancomycin (0.73 DDD/1000 PD). Data recorded show that 46.24% of the total antibiotics were consumed prophylactically.

The total number of infections in the whole hospital was 2870 and the most frequently diagnosed were those of the urinary tract (*n* = 1031), followed by wounds (*n* = 947) and respiratory tract infections (*n* = 651). One third of the total number of pathogens of surgical inpatients were isolated from wounds (51.5%), urinary tract (39.72%), fluids (4.92%) as can be seen in Table 3. A total of 934 strains of pathogens were isolated in the surgical wards. The most commonly isolated pathogen was *E. coli* (33.83%) responsible for most of the urinary tract, skin and soft tissue infections (Table 4). 

The second most frequently isolated pathogen was *S. aureus* (14.66%), a common cause of skin and soft tissue infections, in almost the same proportions as *Escherichia coli*. *Enterococcus faecalis* was the third most frequently isolated pathogen, firstly from urinary tract infections and secondly from wounds. *Enterobacter* spp. (9.10%), *Proteus* spp. (5.99%), *Pseudomonas aeruginosa* (5.13%) and *Klebsiella* spp. (4.38%) were isolated from urinary tract infections and wounds, almost in the same ratio. *Morganella* spp., *Citrobacter* spp., *Serratia* spp., *Acinetobacter* spp., *Streptococcus* spp., and *Enterococcus faecium* were isolated in lower percentages. As a partial conclusion, gram negative pathogens were isolated more frequently than the gram-positive ones (65.31% vs. 34.68%).

By analyzing the cumulative antibiogram results for all isolated pathogens, their sensitivity rates are presented in Table 5.

**Figure 2:**
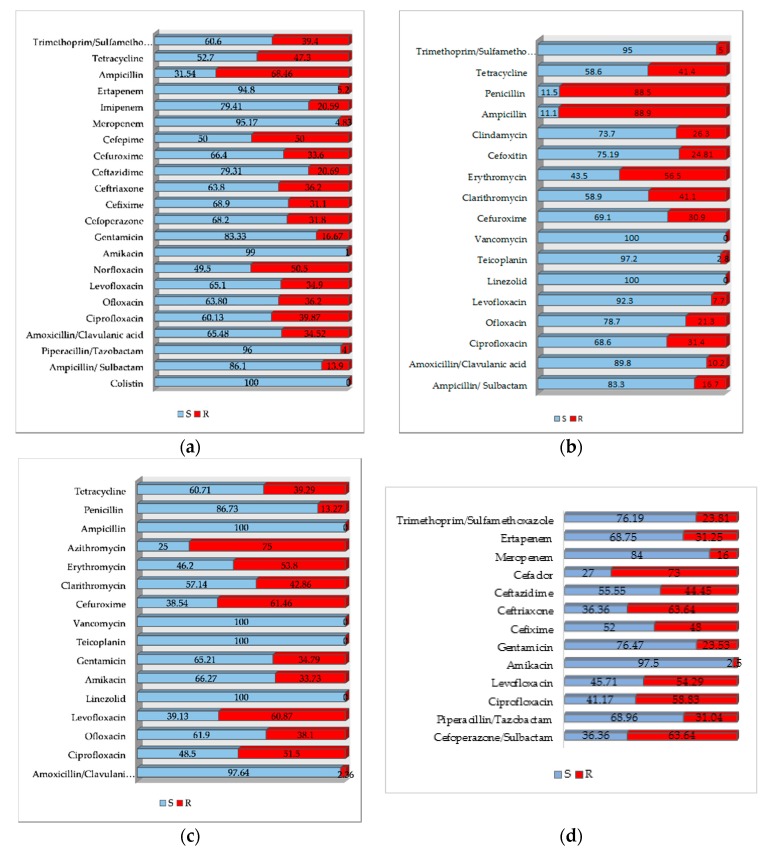
Antibiotics resistance/susceptibility profile for: (**a**) *E. coli*; (**b**) *S. aureus*; (**c**) *E. faecalis*; (**d**) *Klebsiella* spp.

The antibiotic resistance/susceptibility profile for *E. coli*, *S. aureus* and *E. faecalis strains* are shown in Figure 2a–d.

**Figure 3:**
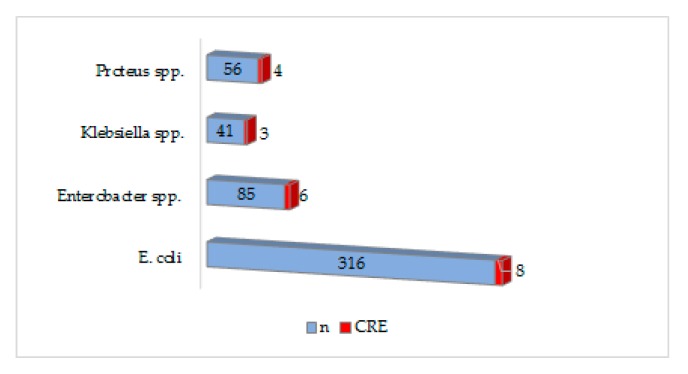
Prevalence of carbapenem resistance in Enterobacterales.

The percentages of MRSA in the surgical departments corresponds to a quarter of all *S. aureus* isolated, while the percentages of VRE is low (6.5%) as well for carbapenem resistant Enterobacterales. Instead, the proportion of extended-spectrum beta-lactamase (ESBL) producing Enterobacterales is higher, especially for *Klebsiella* spp. (19.51%) and *E. coli* (17.72%). All these special phenotypes can be seen in Table 6. As well, the resistance to carbapenems was predominantly expressed by *E. coli* and *Enterobacter* spp. (Figure 3) 

## 3. Discussion

Antimicrobial consumption in Romania is complex and insufficiently explored [10,11,12]. Many published reports have shown high and excessive use. The data provided was only from the community (primary care sector), and the values were 28.5 DDD per 1000 inhabitants and per day in 2015; the same report showed that the most widely used were beta-lactam antibacterial, penicillin and quinolones [7]. Consumption of antibiotics in surgical wards is high, considering both prophylactic and curative treatment, according to updated protocols and procedures, major management objectives of the department, respectively of the hospital [11,12]. Surgical site infections are the third most common type of hospital-acquired infections and, on average, account for 17% of their total, in industrialized countries in recent years [13]. Most of the surgical site infections are caused by endogenous translocation of the patient’s intestinal microbiota, but there is insufficient scientific evidence to determine which groups of antimicrobials are the best for antimicrobial prophylaxis [14]. 

This is a study that explores the antimicrobial consumption and the susceptibility to antibiotics of isolated strains in the surgical wards, for one year, and provides potential measures to improve care locally. Knowing antibiotic susceptibility or resistance of bacteria occurred from surgical site infections has practical importance in optimizing prophylactic antibiotic therapy of surgical procedures and avoiding the selection of multi-resistant pathogens. According to the processed data, the first partial conclusion was a higher antibiotic consumption expressed in DDDs/1000 PD both in the entire hospital and on different wards, including surgical ones as described by other authors [15]. Antibiotic consumption varied between 0.64 and 40.42 DDDs in different surgical departments, explainable by the heterogeneity of ward size and characteristics of the patients’ diseases. Antimicrobials are prescribed mainly for skin, soft tissue and urinary tract infections similar to other data reported in the literature, in accordance with the type of patient population and the studied geographical area [15,16].

More than half of the antibiotics prescribed in surgical wards were ceftriaxone, cefuroxime and ceftazidime; the sensitivity rates were 48.73%, 52.33%, and 68.93%, respectively. The most frequently prescribed antibacterial was ceftriaxone (43.98%). In this study, Enterobacterales (*E. coli*, *Enterobacter* spp., *Klebsiella* spp., *Serratia* spp., *Citrobacter* spp.), on which ceftriaxone is active, were isolated in 57.92% of cases, as can be seen in Table 4, but annual sensitivity rates to ceftriaxone were quite low (48.73%). *E. coli* exhibited lower resistance to cephalosporins (except ceftriaxone), quinolones and carbapenems, as shown in other studies conducted in a different Romanian area [17]. The increased consumption of ceftriaxone could explain the reduced sensitivity of isolated strains and using ceftazidime or cefuroxime could be a better therapeutic option. On the other hand, the most commonly isolated pathogen was *E. coli*, and only 17.72% were ESBL producing, discordant to other studies that showed an increase in the incidence of extended-spectrum β-lactamase-producing *E. coli* worldwide [18]. *E. coli* was responsible for more urinary infections than skin and soft tissue infections, and the therapeutic option was primarily the third generation cephalosporins followed by fluoroquinolones to which strains showed good sensitivity rates, except for ceftriaxone. In the studied hospital, the most prescribed antibiotics were the 3rd generation of cephalosporins (ceftriaxone and ceftazidime); according to guidelines [19], ceftriaxone and ceftazidime are not the first indicated for administration. Results of this research indicated that *E. coli* demonstrated good sensitivity rates to all tested antibiotics except for ampicillin and norfloxacin (Figure 2a). There are other therapeutic options in case of urinary tract infections caused by the *E. coli* or other *Enterobacterales* like trimethoprim/sulfamethoxazole, fosfomycin trometamol, quinolones or first generation of cephalosporins, as recommended by the specific guidelines [19]. 

Gram positive microorganisms (*S. aureus* and *E. faecalis*) were the second and third most frequently isolated strains; they showed good sensitivity to linezolid (100%), teicoplanin (97.01%), vancomycin (96.81%). The slight reduction in susceptibility to vancomycin was found only for a few strains of *E. faecium*. Except for methicillin resistant strains, the best therapeutic option for *S. aureus* is oxacillin; by tracking the consumption of antibiotics expressed as DDD/1000 PD, it is obvious that it has been prescribed for these infections. The overall resistance to amikacin was low (9.5%) and moderated to gentamycin (24.33%). The prophylactic use of gentamycin may be, therefore, justified. 

Third generation cephalosporins were the most-used for perioperative use, as well as in combination with aminoglycoside to cover a wider spectrum. Nevertheless, the cephalosporins are still recommended as prophylactic antibiotic therapy in abdominal surgery. The obtained results indicated that the patients received 3rd generation cephalosporins either alone or in combination with aminoglycosides, taking into account the number of prescribed doses.

Clindamycin is recommended primarily for the treatment of anaerobic infections and in patients with hypersensitivity to penicillin to treat infections caused by susceptible aerobic bacteria as well. It is also used to treat bone and joint infections, particularly those caused by some methicillin-resistant *S. aureus* strains, 24.81% in the present study. The annual sensitivity rates to clindamycin were 68.18%. Anaerobic microorganisms were not isolated in 2017, but clindamycin was the fourth antimicrobial prescribed or a third of the total amount in the hospital. Metronidazole was less prescribed, although some scientists note that the surgical site infection rate was lower with the combination cefazolin (or other cephalosporin) plus metronidazole for contaminated surgeries of gut and gall bladder, in which anaerobic organisms are expected to be present.

The third frequently isolated microorganism from urinary tract infections and wounds was *E. faecalis*. These strains showed good rates of susceptibility to antimicrobials, and its treatment with ampicillin possibly associated with gentamycin explains their consumption. Considering that 88.5% of staphylococci were penicillinase producers (Figure 2b), penicillin use is warranted only if they have been prescribed for the treatment of infections produced by *E. faecalis*. *E. faecium* presented therapeutic issues for which the alternative is linezolid, tetracycline and vancomycin (if not resistant to vancomycin), but the prescriptions of these were low corresponding to a low number of these special strains.

The ESBL-producing strains are particularly resistant to all penicillin, to cephalosporins and to aztreonam. Furthermore, they are often cross-resistant to other antimicrobials, such as trimethoprim/sulfamethoxazole, quinolones. This combination of resistance can significantly change the course and outcomes of infections, whether they are from the community or hospital settings. In these special strains, the therapeutic options are penicillin and beta-lactamase inhibitors combinations; in the present study, they were less prescribed corresponding to their reduced percentage and good cumulative sensitivity rates for (ampicillin/sulbactam, amoxicillin/clavulanic acid, piperacillin/tazobactam). 

The acquisition of carbapenemase genes is one of the most frequent mechanisms for the gram-negative bacteria to present resistant to carbapenems. There are three classes of carbapenemases described: Class A carbapenemase, class B metal-enzymes, and class D enzymes [20,21]. In a Romanian study on 1040 isolates from surgical wards, the prevalence of ESBL production was 32.3% and 23.53% of carbapenemase producing strains only for *Klebsiella* spp. [22]. In this study, ESBL-producing strains were mainly *Klebsiella* spp. and *E. coli* and resistance to carbapenems was predominantly expressed by *Enterobacter* spp. and *Klebsiella* spp.

According to a WHO report in 2013, among EU-countries the highest prevalence of *K. pneumoniae* resistant to the third generation cephalosporines (88.5%) and a very high prevalence of *K. pneumoniae*, *P. aeruginosa* and *A. baumannii* resistant to carbapenems (31.4%, 44.6% and 92.1%, respectively) was already described. In addition, the percentages of multi drug resistant (MDR) pathogens relative to the total number of isolates were higher on the surgical wards where the urinary tract, gastrointestinal tract and skin and soft tissue infections were the most frequently diagnosed [23,24]. By contrast, in the hospital in the study, MDR pathogens have been particularly encountered in the intensive care unit, where the main etiologic agent of respiratory infections was *A. baumannii* [25]. 

Screening procedures for the detection of MRSA are recommended in all patients at high risk of MRSA carriage, especially those from intensive care, trauma and orthopedic surgery, where Staphylococcus strains account for most surgical site infections. A quarter of the *S. aureus* strains, the second etiologic agent of the infections on the surgical wards, were resistant to methicillin. Treatment of the infections produced by these strains can be done with vancomycin and linezolid, but they were less prescribed (Table 2). If the rate of resistance to methicillin of *S. aureus* will increase this will be followed by higher use of glycol-peptides and resistance to them.

As acknowledged, carbapenems are recommended over other types of antimicrobials in treating invasive or life-threatening infections because of their broad-spectrum abilities to include anaerobes. The most prescribed was Meropenem showing sensitivity rates of 88.64%; Ertapenem, less prescribed in comparison with Meropenem, presented a better sensitivity in 89.62% of the tested strains. Both are useful in the treatment of a variety of infections (complicated intra-abdominal and skin infections, community-acquired and nosocomial pneumonia, complicated urinary tract infections, meningitis and febrile neutropenia). The difference in susceptibility is the result of more frequent use of Meropenem. Imipenem and cilastin were less used, although showed sensitivity in 60.56% of the tested gram-negative strains.

Fluoroquinolones were the third class of antibiotics prescribed in surgical wards; the preferred agent was ofloxacin followed by ciprofloxacin, levofloxacin. Less prescribed were moxifloxacin and norfloxacin. Cumulative sensitivity rates were 63.61% for ofloxacin, 69.62% for levofloxacin, 55.23% for ciprofloxacin. The resistance to quinolones has been reported to emerge as a result of treatments with ciprofloxacin. In the current study, a ciprofloxacin resistance of 44.76% was found, possibly as many prescriptions for urinary infections or as prophylactic therapy.

In Romania the cases are treated according to the internal guides/protocols of the hospitals. The infectious specialist is asked for advice in serious cases (resistance to drugs/antibiotics, respectively ineffective drug treatment, or poor patient’ evolution). In the studied hospital, if an etiology with a drug-resistant strain is assumed or found, Vancomycin is given for gram positive and Cefoperazone + sulbactam for gram negative; also, the strain test for the antibiotic administered is mandatory.

A major component in the medical audits is represented by the analysis of antimicrobial consumption, which is essential when evaluating, monitoring and making the needed modifications in doctors’ treatment schemes, in order to obtain cost-effective and rational medical care. Results of this research indicate the necessity of implementing and following the programs that control microbe resistance in inpatients; each hospital must enact priority measures to control the emergence of antibiotic-resistant bacteria, including control of overuse. Ideal management of antimicrobial use consists of the need for physicians to recommend for patients the most harmless treatment, using the lowest-priced antibiotic, for the shortest amount of time, to prevent or cure an infection. In the literature, four strategies are highlighted in order to achieve the goals above: Cyclic or rotary use of antimicrobials; restrictive and selective control of some antimicrobial agents; use of all antimicrobials in a rationally way; in order to prevent resistance emergence, the use of the combined antimicrobial treatment, where necessary [25]. 

The limitations of this study are the short observation period (one year) and the impossibility to differentiate among every single patient and its comorbidities, as well as distinguishing community —from the hospital—acquired pathogens. However, this work is a comprehensive analysis of the use of the most important antibiotics for the treatment of different infections and corresponding antimicrobial resistance across multiple departments within one institution. All clinical samples isolated across surgical departments were included, and the standardized ATC/DDD system was used. The study, therefore, may have important relevance for local clinical practice.

## 4. Materials and Methods 

This retrospective study was performed at the County Clinical Emergency Hospital of Oradea (Oradea, Romania) among surgical inpatients admitted between 1st January and 31 December 2017. The hospital is an acute care university-affiliated hospital, with 861 acute care beds, 278 (32.28%) belonging to the surgical departments. For this research, data were collected from general surgery, burns and plastic surgery, thoracic, cardiac and vascular surgery, neurosurgery, urology, orthopaedics, oral and maxillofacial surgery, otorhinolaryngology, and ophthalmology. 

Information from the patients’ medical records and Whonet 5.6 software were used to explore the number, type of pathogens and susceptibility to antimicrobials. The identification of the pathogens was performed by the growing cultural characters, standard biochemical methods and matrix-assisted laser desorption/ionization (MALDI). The antibiotic susceptibility was determined using the VITEK 2 compact system and Kirby Bauer method according to the guidelines of the Clinical and Laboratory Standards Institute (CLSI). Antimicrobial resistance or susceptibility monitoring was performed using the annual summary report (cumulative antibiogram), reporting only the percentage of susceptible strains. CLSI M39-A2 recommends an annual analysis or a limit of at least 30 isolates of a species collected over a longer period mentioning in the report the period [26]. The result was interpreted and, as the case was, the confirmation test of the resistance phenotype for gram negative was performed (for instance, double-disk synergy test and/or combination disk test—cefotaxime, ceftazidime and their combination with clavulanic acid, for ESBL phenotypic confirmation). 

Data on each of the prescribed antibiotics were extracted from the computerized records of the hospital pharmacy. The prescription pattern of antimicrobials was analyzed using the DDD methodology (ATC/DDD Index 2018) [8]. The number of administrated units (vials, bottles, capsules) was converted into the number of daily define doses (DDD), as recommended by WHO. Antimicrobial density was expressed as DDD per 1000 patient-days for each antimicrobial, in order to control the population size. The annual number of patient-days was provided by the admission department of the hospital. One patient-day is defined as the provision of accommodations and services for a single patient on a single day. Patient-day data, together with DDDs, expressed antibiotic consumption as defined daily doses per 1000 patient days (DDDs/1000 PD).

All the antibiotics prescribed in the therapy and prophylaxis of hospital acquired infections produced by gram-negative and gram-positive bacteria, defined as antimicrobials for systemic use or group J01 of the WHO Anatomical Therapeutic Chemical (ATC) classification system [8], and excluding anti tuberculous drugs (rifampicin), topical antibiotics, rifaximin, metronidazole, antifungals and those not purchased in the described institution at that time (fosfomycin, ticarcillin, ticarcillin + tazobactam, tobramycin). Metronidazole is commonly used in bowel surgery and must be given 2–4 h preoperatively. Its consumption was analyzed separately, as it did not show sensitivity like topical antibiotics. Only the doctors/surgeons have the authority to start antibiotic therapy. According to the policies of the hospital, for prophylaxis it is administered a single intravenous dose of antibiotic before surgery in the case of clean and clean-contaminated wound procedures; in the case of abdominal surgery, it is administered Metronidazole.

The positive pathological samples were sputum, fluids (pleura, peritoneal, pericardial), lesions fluids, wounds, catheters, urine, blood. Most patients are tested after 5–6 days of antibiotic treatment or before discharge to evaluate its evolution (the samples are collected from patients after the completion of the antibiotic course and culturing); if the evolution of the case is not favorable, the collection of new biological samples is repeated. The postoperative infection was coded according to WHO, ICD codes (T81.4 Infection following a procedure) [27]. 

### Statistical Analysis

Descriptive statistics were applied to the collected data using Microsoft Excel 2010 software. Results are expressed in percentages and averages.

## 5. Conclusions

This research highlights the antimicrobials prescribed mainly for skin, soft tissue and urinary tract infections, as well as the dominance of gram-negative bacteria as etiologic agents. Cephalosporins continued to remain the main option in antibiotic therapy, both in the hospital and surgical wards, because of their broad spectrum of activity and increased prevalence in gram-negative pathogens. The most commonly prescribed medicine was ceftriaxone, but its sensitivity rate was low. 

The selection and duration of antibiotic administration can be improved. Based on, and considering the recorded results and data, new priority measures must be implemented in order to control the resistance of the microbes in inpatients, and the surveillance of the antimicrobial use, especially by controlling the over-use. Improving perioperative prophylaxis and infections therapy in the mentioned surgical departments will be a priority in the future for preventing the increasing prevalence of MDR pathogens. 

## Figures and Tables

**Table 1:** Antibiotics prescribed by classes.

Antibacterial Name	DDD/1000 PD				Type of Pathogen Treated: Gram
	Surgical Wards		Total Hospital		
	No.	%	No.	%	
Cephalosporins (including combinations with inhibitors)	311.61	65.02	1183.89	54.30	− and +
Fluoroquinolones	36.31	7.57	239.7	10.99	− and +
Aminoglycosides	27.79	5.79	167.0	7.65	− and +
Penicillin and beta-lactamase inhibitor	65.81	13.73	234.6	10.76	− and +
Carbapenems	6.22	1.29	119.1	5.46	−
Polymyxins (Colistin)	0.49	0.10	55.7	2.55	−
Lincosamide (Clindamycin)	24.34	5.07	64.2	2.94	+
Glycol-peptides	1.24	0.25	49.3	2.26	+
Tigecycline	0.20	0.04	26.1	1.19	+
Linezolid	0.40	0.08	19.7	0.90	+
Macrolides	1.84	0.38	11.9	0.54	+
Tetracycline (Doxycycline)	0.25	0.05	4.1	0.18	−
Metronidazole	2.68	0.55	4.89	0.22	− and +

**Table 2:** Utilization pattern of antibiotics expressed as DDD/1000 PD in the surgical wards.

Antibacterial Name	ATC Code	DDD/1000 PD	
		No.	%
Ceftriaxone	J01DD04	211.92	43.98
Cefuroxime	J01DC02	54.81	11.37
Ceftazidime	J01DD02	30.91	6.41
Clindamycin	J01FF01	24.22	5.03
Gentamycin	J01GB03	22.79	4.73
Ampicillin	J01CA01	19.47	4.04
Oxacillin	J01CF04	19.21	3.99
Ofloxacin	J01MA01	18.81	3.90
Ciprofloxacin	J01MA02	12.08	2.51
Benzylpenicillin	J01CE01	11.39	2.36
Cefoperazone	J01DD12	10.75	2.23
Amoxicillin + clavulanate	J01CR02	8.35	1.73
Amoxicillin	J01CA04	6.01	1.25
Amikacin	J01GB06	5.14	1.07
Levofloxacin	J01MA12	4.32	0.90
Meropenem	J01DH02	3.28	0.68
Cefoperazone + sulbactam	J01DD62	2.98	0.62
Metronidazole	J01XD01	2.68	0.56
Ertapenem	J01DH03	2.57	0.54
Clarithromycin	J01FA09	1.63	0.34
Piperacillin + tazobactam	J01CR05	1.60	0.33
Cefaclor	J01DC04	1.55	0.32
Moxifloxacin	J01MA14	0.98	0.20
Ampicillin + sulbactam	J01CR01	0.83	0.17
Vancomycin	J01XA01	0.73	0.15
Norfloxacin	J01MA06	0.72	0.15
Colistin	J01XB01	0.46	0.10
Teicoplanin	J01XA02	0.39	0.08
Imipenem/cilastin	J01DH51	0.38	0.08
Linezolid	J01XX08	0.30	0.06
Cefixime	J01DD08	0.18	0.04
Doxycycline	J01AA02	0.18	0.04
Azithromycin	J01FA10	0.12	0.03
Tigecycline	J01AA12	0.12	0.02

**Table 3:** Number and frequency (%) of types of infection.

Type of Infection	Total Infections (*n* = 2870)	Surgical Wards (*n* = 934)	
		*n*	%
Respiratory tract	651	26	2.78
Urinary tract	1031	371	39.72
Wounds	947	482	51.50
Blood	58	3	0.32
Catheter	46	6	0.64
Fluids (pericardial, peritoneal)	137	46	4.92

**Table 4:** Number and frequency (%) of the isolated pathogens.

Strain	Total (No./%)	Respiratory Tract	Urinary Tract	Wounds	Blood	Catheter	Fluids
*Staphylococcus aureus*	137/14.66	6	8	118	2	0	3
*Staphylococcus coagulase negative*	41/4.38	2	0	36	1	2	0
*Enterococcus faecalis*	123/13.16	0	81	42	0	0	0
*Enterococcus faecium*	14/1.49	0	7	5	0	2	0
*Staphylococcus pyogenes*	2/0.21	1	0	1	0	0	0
*Streptococcus* spp. (*B*, *F*, *G*, *viridans*)	7/0.74	0	0	7	0	0	0
*Escherichia coli*	316/33.83	3	164	114	0	0	35
*Enterobacter* spp.	85/9.10	4	36	41	0	0	4
*Proteus* spp.	56/5.99	2	21	33	0	0	0
*Klebsiella* spp.	41/4.38	2	18	20	0	0	1
*Acinetobacter* spp.	21/2.24	4	0	14	0	2	1
*Pseudomona aeruginosa*	48/5.13	2	16	28	0	0	2
*Serratia* spp.	21/2.24	0	9	12	0	0	0
*Morganella* spp.	19/2.93	0	8	11	0	0	0
*Citrobacter* spp.	3/0.32	0	3	0	0	0	0
Total		26	371	482	3	6	46

**Table 5:** Sensitivity rates of tested antibiotics (according to cumulative antibiogram).

Antibacterial Name	ATC Code	Cumulative Sensitivity Rates (%)	Isolates: Gram
Linezolid	J01XX08	100	+
Vancomycin	J01XA01	98.84	+
Colistin	J01XB01	98.33	−
Teicoplanin	J01XA02	97.46	+
Amikacin	J01GB06	90.5	−
Ertapenem	J01DH03	89.62	−
Piperacillin/Tazobactam	J01CR05	89.58	− and +
Meropenem	J01DH02	88.64	−
Ampicillin/Sulbactam	J01CR01	86.24	− and +
Amoxicillin/Clavulanic acid	J01CR02	78.77	− and +
Gentamycin	J01GB03	75.66	−
Cefoperazone/Sulbactam	J01DD62	71.59	−
Ceftazidime	J01DD02	68.93	−
Clindamycin	J01FF01	68.18	+
Ofloxacin	J01MA01	63.61	− and +
Cefoperazone	J01DD12	62.75	−
Imipenem/cilastin	J01DH51	60.56	−
Cefixime	J01DD08	60.2	−
Trimethoprim/Sulfamethoxazole	J01EE01	69.69	− and +
Levofloxacin	J01MA12	59.62	− and +
Clarithromycin	J01FA09	57.37	+
Cefaclor	J01DC04	55.31	−
Ciprofloxacin	J01MA02	55.23	− and +
Ampicillin	J01CA01	52.9	− and +
Cefuroxime	J01DC02	52.33	− and +
Tetracycline	J01AA07	51.5	−
Cefepime	J01DE01	51.42	−
Ceftriaxone	J01DD04	48.73	-
Norfloxacin	J01MA06	47.52	- and +
Penicillin	J01CE01	41.63	+
Erythromycin	J01FA01	39.75	+
Azithromycin	J01FA10	36.84	+
Amoxicillin	J01CA04	30.95	− and +

**Table 6:** The frequency of strains with different phenotypes of resistance.

Strain	*n*	ESBL	CRE	MRSA	VRE
		(No./%)			
*E. coli*	316	56/17.72	8/2.5	−	−
*Enterobacter* spp.	85	11/12.94	6/7.05	−	−
*Klebsiella* spp.	41	8/19.51	3/7.31	−	−
*P. aeruginosa*	48	5/10.41	2/4.16	−	−
*Proteus* spp.	56	6/10.71	4/7.14	−	−
*S. aureus*	137	−	−	34/24.81	−
*E. faecalis*	123	−	−	−	8/6.5
